# What does it mean to have an impairment?

**Published:** 2013

**Authors:** Gertrude Oforiwa Fefoame

**Affiliations:** Africa Social Inclusion Advisor for Sightsavers, an international charity which works in low- and middle-income countries to restore sight and support people who are irreversibly blind. She spends her time advocating and providing technical support for the inclusion of people with disabilities in eye care and other development projects.

**Figure:**
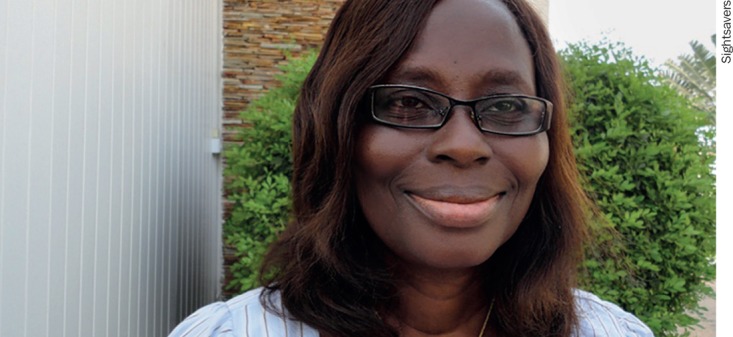
Gertrude Oforiwa Fefoame

‘When I was ten years old and in school, I realised I couldn't read from the blackboard like the other children in my class. My family took action immediately, and I was seen by an ophthalmologist at the most advanced eye clinic in Ghana at the time. I was referred to an optometrist and given spectacles, but I needed a new prescription every three months. Eventually we were told that there were no other reading glasses that could help.

‘Even though I grew up in the vicinity of the first school for the blind in Ghana, I remained in my mainstream school and continued to a mainstream secondary school at the age of 13. By the time I was 14, it was really difficult for me to read textbooks: I could only read large print and my own handwriting. I learned mainly by listening and also working with my classmates, who gave me support as we studied and did our homework. Some teachers would offer extra help after the class, and others were willing to read what they were writing on the board so I could hear and follow. But it was not a formal low vision service. I didn't know that any existed as low vision students at the School for the Blind then were all learning like blind students.

‘Later, when I had finished school, I met one of my teachers, and he explained that the headmaster of the school had received some exposure to special needs education and gave the teachers hints on how they could support me. Because I was not involved in the discussion and did not know about my rights then, I didn't know I had the right to demand such services. I didn't know that what those staff members did for me was not charity, but their responsibility. This meant I didn't feel I was able to ask for the additional support that I really needed in school.

‘At the hospital, when they could no longer improve my vision or even prevent it from getting worse, nobody explained to me what the condition was and what I should expect in the future. I am not sure whether my relatives had a better understanding than I had, but they didn't tell me much. It was also not normal for a child in my culture to ask too many questions.

‘When it came to my final examinations, although the school applied for questions in large print, two weeks before the examination information reached me that the examining board could not provide this. Fortunately, my biology teacher had an idea – I could use a hand magnifying lens, like the ones we used to examine specimens! Although I could see only a few letters at a time, as it was such as small lens, I was able to read the exam questions. I still have the lens today although it is no longer of use!

**‘I learned mainly by listening and working with my classmates’**

‘Soon after I left school, there was an advert in the paper about teachers who could be trained to support people with visual impairment. My uncle saw this and investigated – he found out that I could go to the school for the blind where I could learn to read and write Braille, so I could continue my education.

‘The admission form for the school for the blind had to be signed by an ophthalmologist, to certify that I needed such a service. My ophthalmologist, who I've been with for many years, said he thought it was a good idea for me to go, but he couldn't say why he had never suggested this before!

‘When I went to the school for the blind, which was near where I grew up, the people I knew in the area reacted very differently to me – even though my vision hadn't changed. They were sad, would say how sorry they were for me, and spoke with such pity!

‘I was visited at the school by a lady who is blind and who was already enrolled at the teacher training college. I listened to her talk about her experiences and then I knew that I had a future.

‘Over the next two decades, I qualified as a teacher, then specialised in special needs education, followed by a degree course in education. I taught psychology, counselling, and special needs education at the teacher training college.

‘Maturing as a person with a visual impairment was very difficult – society didn't make it easy. Thinking back, I knew that the way people reacted to me when I went to the school for the blind was wrong but I didn't know what to do about it. I struggled with this and similar concerns for many years.

‘Then, in the early 1990s, things changed when I attended a workshop initiated by the World Blind Union's Institutional Development Programme (IDP). IDP is an international capacity building programme for organisations of and for the blind mainly in Africa, and is sponsored by Sightsavers and Perkins International. At the workshop, I realised that continued advocacy and awareness raising would be required to address the challenges faced by individuals and organisations for the blind. What stood out for me was that everybody had a role; you could initiate change from wherever you were and engage others to join you. It had a great impact on me and encouraged me. So I strengthened my participation in the organisations of persons with disabilities at national, regional (Africa) and global levels, and served in different leadership positions.

‘Whenever one is able to push disability behind and move on with life, those with positive thoughts see the person and not the disability. Sometimes, my friends forget that I cannot see – it is because they see me, and not my visual impairment. Everybody in society can be like this – particularly if we start by educating our children that people with disabilities are just the same as everyone else.’

**If I could choose…**

**Eye care practitioners would be careful how they promoted the restoration of sight**

‘Don't create the impression that, if vision cannot be restored, that it is the end of that individual. Right from the beginning, eye care practitioners should say: “I know you are managing, but some of the challenges you are facing can be limited.” Then, if the operation is not successful, they can say: “Well, you remember the conversation we had, about how well you were managing, this is what we need to continue and strengthen. There are also these services I can refer you to for more support…”’

**Eye care practitioners would talk to people with disabilities, instead of their guides or carers**

‘People think the person who walks in with you knows more about you than you yourself, even if you are an adult. It is usually because there is no eye coordination, and sighted people feel more comfortable talking to people with whom they can link eyes (make eye contact). This should be explained to people not just in the eye profession, but across the board. So, more awareness within the health training, and refresher courses around disability, are needed.’

**Pharmacists would ensure people with disabilities understood how to use their medicines**

‘The ideal would be the provision of a Braille label to those who can read it. For those who can't, pharmacists can help them to examine the package, and perhaps the content, so they are able to identify the correct dosage. This could also be provided by the Support Services Department, which (ideally) every hospital should have.’

**People promoting eye health would ensure that people with disabilities also get the information they need**

‘If you are giving a health talk, or doing health education in a community, check that people with different impairments are also there to listen. There will certainly be several people with impairments in the community – you are responsible for ensuring that they also hear your message. Don't rely on others to tell them. Remember, also, that people with a visual impairment cannot read posters.’

**People working in eye clinics would value all patients**

‘People coming for eye care often have some visual disability already. They will experience fear, anxiety, and confusion, as well as worries about the costs. So when they come to the clinic, and the receptionist – whoever is doing the papers – is harsh, then it gets much more difficult. If the people you meet are warm and friendly, it is much easier. The way people working in eye clinics treat people with impairments is very important: be polite, understanding, and encouraging. Despite the fact that there is so much work that needs to be done, the eye team have to be very professional – this should be part of their training.’

**People with disabilities would be included in the health sector**

‘Eye health programmes need to include people with disabilities in all aspects of health promotion, blindness prevention, and eye care delivery. For example, people with disabilities would make excellent counsellors for people who have become disabled, as they are good role models and mentors.’

**Counselling would empower people with impairments**

‘This is the type of counselling that deals with the inner awareness and self-actualisation of the person – it is about that person reaching their full potential, disabled or not. Yes, it's good to tell people that there are services available for them, but this is about their head or heart, which is telling them that the world has come to an end. That is the perspective good counselling tries to change. Counsellors must say to people: “This is not your end”, and talk to them about others who are doing well and even excelling in life, despite their impairments.

‘Counsellors must also empower people as individuals: tell them they have a right to ask for the assistance they need, that they have a right to participate. Help them to develop assertiveness and confidence in themselves as an individual – a platform which every person needs to develop and grow. This is when people with impairments can grow from strength to strength.’

